# International Medical Annual for 1895

**Published:** 1895-01

**Authors:** 


### International Medical Annual for 1895.

E. B. Treat, Publisher, New York, has in press for early publication the 1895 *International Medical Annual*, being the thirteenth yearly issue of this eminently useful work. Since the first issue of this one-volume reference work, each year has witnessed marked improvements, and the prospectus of the forthcoming volume gives promise that it will surpass any of its predecessors. It will be the conjoint authorship of thirty-eight distinguished contributors and specialists, from America, England, and the Continent. It will contain the progress of Medical Science in all parts of the world, together with a large number of original articles and reviews by authors on subjects with which their scientific reputation is identified. In short, the design of the book is to bring the practitioner into direct communication with those who are advancing the Science of Medicine, so he may be furnished with all that is worthy of preservation as reliable aids in his daily work. Illustrations in black and colors will be freely used in elucidating the text. A most useful investment for the medical practitioner. The price remains the same as heretofore, $2.75.

E. B. Treat, Publisher, also has in press the following books, which will be issued early in 1895:

